# Subclinical Mastitis in Selected Bovine Dairy Herds in North Upper Egypt: Assessment of Prevalence, Causative Bacterial Pathogens, Antimicrobial Resistance and Virulence-Associated Genes

**DOI:** 10.3390/microorganisms9061175

**Published:** 2021-05-29

**Authors:** Ahmed H. Abed, Ahmed M. S. Menshawy, Mohamed M. A. Zeinhom, Delower Hossain, Eman Khalifa, Gamal Wareth, Mohamed F. Awad

**Affiliations:** 1 Bacteriology, Mycology and Immunology Department, Faculty of Veterinary Medicine, Beni-Suef University, Beni-Suef 62511, Egypt; 2 Department of Veterinary Medicine (Infectious Diseases), Faculty of Veterinary Medicine, Beni-Suef University, Beni-Suef 62511, Egypt; elmenshawy81@yahoo.com; 3 Food Hygiene and Control Department, Faculty of Veterinary Medicine, Beni-Suef University, Beni-Suef 62511, Egypt; m.zeinhom@vet.bsu.edu.eg; 4 Department of Medicine and Public Health, Faculty of Animal Science and Veterinary Medicine, Sher-e-Bangla Agricultural University, Sher-e-Bangla Nagar, Dhaka 1207, Bangladesh; delowervet@sau.edu.bd; 5 Department of Microbiology, Faculty of Veterinary Medicine, Matrouh University, Matrouh 51511, Egypt; khalifa.eman@alexu.edu.eg; 6 Friedrich-Loeffler-Institut, Institute of Bacterial Infections and Zoonoses, Naumburger Str. 96a, 07743 Jena, Germany; gamal.wareth@fli.de; 7 Faculty of Veterinary Medicine, Benha University, Moshtohor, Toukh 13736, Egypt; 8 Department of Biology, College of Science, Taif University, P.O. Box 11099, Taif 21944, Saudi Arabia; m.fadl@tu.edu.sa

**Keywords:** subclinical mastitis, antimicrobial resistance, *E. coli*, *Staphylococcus aureus*, NAS, Streptococci, virulence genes, Egypt1

## Abstract

Mastitis is a significant disease affecting dairy cattle farms in Egypt. The current study aimed to investigate the prevalence and major bacterial pathogens causing subclinical mastitis (SCM) in three bovine dairy herds, with a history of SCM, at three Governorates in North Upper Egypt. The antimicrobial resistance profiles and specific virulence-associated genes causing bovine SCM were investigated. One thousand sixty-quarter milk samples (QMS) were collected aseptically from 270 apparently healthy cows in three farms and examined. The total prevalence of SCM was 46% and 44.8% based on California Mastitis Test (CMT) and Somatic Cell Count (SCC), respectively. Bacteriological examination of CMT positive quarters revealed that the prevalence of bacterial isolation in subclinically mastitic quarters was 90.4% (26 and 64.3% had single and mixed isolates, respectively). The most frequent bacterial isolates were *E. coli* (49.8%), *Staphylococcus aureus* (44.9%), streptococci (44.1%) and non-*aureus* staphylococci (NAS) (37.1%). Antimicrobial susceptibility testing of isolates revealed a high degree of resistance to the most commonly used antimicrobial compound in human and veterinary medicine. Implementation of PCR revealed the presence of *mec*A and *bla*Z genes in 60% and 46.7% of *S. aureus* isolates and in 26.7% and 53.3% of NAS, respectively. Meanwhile 73.3% of streptococci isolates harbored *aph*(3’)-IIIa gene conferring resistance to aminoglycosides and *cfb* gene. All *E. coli* isolates harbored *tet*A gene conferring resistance to tetracycline and *sul*1 gene conferring resistance to sulfonamides. The *fim*H and *tsh* genes were found in 80% and 60%, respectively. A significant association between the phenotypes and genotypes of AMR in different bacteria was recorded. The presence of a high prevalence of SCM in dairy animals impacts milk production and milk quality. The coexistence of pathogenic bacteria in milk is alarming, threatens human health and has a public health significance. Herd health improvement interventions are required to protect human health and society.

## 1. Introduction

Bovine mastitis is a significant challenge globally threatening the dairy industry, affecting both the quantity and quality of milk and resulting in substantial economic losses [1,2]. Mastitis is caused by multi-etiological agents, including various environmental and microbial predisposing factors [2]. In the USA alone, mastitis causes annual losses in the dairy industry, reaching 1.7 billion US dollars a year [3]. Mastitis is divided into two main types, clinical (CM) and subclinical (SCM). Clinical mastitis is recognized by physical, chemical and microbiological alteration found in milk, such as changes in color and consistency of the udder, alteration in milk contents and presence of pathogenic microorganisms [4]. Subclinical mastitis (SCM) is an asymptomatic disease characterized by increased milk somatic cell count and can only be diagnosed by laboratory tools using special tests such as the California mastitis test (CMT), Whiteside test, Somatic cell count (SCC) and Electrical conductivity test [5,6]. It is worth mentioning that SCM causes more than 3–4 times economic losses as compared to CM, and milk production decreases by 17.2% in SCM without the occurrence of any obvious clinical signs [7].

Additionally, sub-clinically mastitic cows should be regarded as a risk for disseminating mastitis pathogens within and between dairy herds [3]. Mastitis could be caused by more than 135 types of microbial pathogens. However, significant pathogens associated with SCM are *E. coli*; non*-aureus* staphylococci (NAS); *Streptococcus* spp., especially *S. agalactiae* and *S. dysgalactiae*; and *S. aureus* [6]. Identification and isolation of various pathogens causing mastitis enable appropriate choices for antimicrobial therapy and proper preventive mastitis management [8,9]. Nowadays, antimicrobial agents are widely used to prevent and control mastitis and other diseases affecting dairy herds [9]. Therefore, the dependency of dairy farms on antimicrobial agents is a commonly widespread phenomenon resulting in many serious problems concerned with the causative agents’ pathogenicity and public health [3].

The excessive use of antimicrobial compounds in veterinary medicine might develop antimicrobial resistance (AMR) in many pathogenic bacteria [3,8,9]. In Egypt, mastitis is widely spread among dairy farms. Mycotic mastitis exists at higher prevalence among sheep flocks due to a lack of antiseptics [10]. Recently, subclinical mastitis due to *S. aureus* [11], *Streptococcus* spp. [12] and *E. coli* [13] was reported in dairy cow herds and was the predominant cause of clinical and subclinical mastitis in goats [14]. *Enterococci* and ESBL-producing *E. coli* harboring different resistance gens were isolated from milk of bovine mastitis cases [15]. Several pathogenic microorganisms have been isolated from bovine milk with mastitis [15,16,17,18]. However, still, the knowledge on subclinical mastitis is rare. Therefore, this study was carried out to determine the prevalence of SCM in three dairy bovine herds in North Upper Egypt and the prevalence of major bacterial pathogens contributing to SCM, as well as to determine the AMR profile and virulence-associated genes in MDR bacterial strains causing SCM.

## 2. Materials and Methods

### 2.1. Milk Samples Collection

A total of 270 apparently healthy cows from three dairy herds located in the North-Upper Egypt governorates (Beni-Suef, El-Fayoum and Giza) were sampled between March and September 2020. The three farms were selected because they are the largest bovine dairy farms that has a history of SCM in each governorate. Cows mainly were between the 2nd and 5th seasons of lactation after the 2nd−7th months of calving. Before sampling, approval was obtained from the Ethical Committee at the Dean’s office at the faculty of veterinary medicine, Beni-Suef University, Egypt (code, BSU/0155/26122019), and permissions from the farm owners were obtained. One sample from each quarter was targeted. A total of 1060 quarter milk samples (QMSs) were collected aseptically from the cows, and the missed twenty QMS were due to presence of a total of twenty non-functioning quarters in cows under study as they were blocked due to the previous history of mastitis. Each udder was washed and carefully dried with a clean towel. Then, each teat was swabbed with 70% ethyl alcohol; first jets of milk were avoided; then, 15–20 mL of mid-stream milk was collected into a sterile screw-capped McCartney bottle. The milk samples were kept in ice containers immediately. They were sent to the laboratory of the Department of Bacteriology, Mycology and Immunology at Faculty of Veterinary Medicine, Beni-Suef University, Egypt, with a minimum of delay for SCC and bacteriological examination.

### 2.2. California Mastitis Test (CMT)

It is a simple cow-side test that has been done on the farm just after taking the QMS for SCC and Bacteriological examination, Equal volumes (2 mL) of each QMS and CMT reagent (4% “detergent” alkyl aryl sulphonate+ Bromcresol purple “pH indicator”) or mastitis indicator test kit (Frieso-test) obtained from Impfstoff work Friesoythe Gmbh-(Germany) were thoroughly mixed in a black plastic paddle cup. The mixture was made in a circular motion by gentile swirling of the paddle for about 5–10 s, and then, results were classified into four scores as follows: negative (−), the mixture remained liquid with no tendency to form precipitate; weak positive (+), a distinct precipitate was formed, but with no tendency to gel formation; distinctive positive (++), the mixture thickened immediately with some suggestion of gel formation; strong positive (+++), a distinct gel is formed with a tendency to adhere to the paddle bottom, and during swirling, a distinct central peak was formed. Cows with at least one positive quarter with a score of (+) or more were considered positive for SCM. Those cows were targeted for milk sample collection for further laboratory analysis.

### 2.3. Somatic Cell Count (SCC)

The collected QMSs were examined for SCC automatically using a Bently Soma Count 150 ( Bentley Instrument, Chaska, MN, USA) to detect any possible variation. The samples were warmed in a water bath at 40 °C for 5 min and then mixed autonomically before the autonomic reading of SCC by Bentley Soma count for dispersion of fat globules [19]. The SCC measures the number of WBCs such as neutrophils, macrophages, eosinophils and lymphocytes, and different types of the mammary gland’s epithelial cell in milk that increased in case of SCM. Individual QMSs showing an SCC ≥ 2.50 × 10^5^ cells/mL were considered positive for SCM, according to Romero et al. [20].

### 2.4. Milk Scanning Using a Milk Scanner Analyzer

The fresh chilled collected QMSs were examined to measure all milk constituents such as protein, lactose, fat, solid not fat (SNF), ash and water using MilkoScanTM Milk Analyzer (Foss, Hilleroed, Denmark). Approximately 6 mL of fresh chilled milk samples was put in the apparatus’s specific container, and the sensitive measuring SS loop was inserted inside the milk sample. The automatic electronic scanning was obtained within around 40–50 s. Cleaning of the specified container was obligatory between each sample using the same loop and cleaning step of the program.

### 2.5. Bacteriological Examination of Milk Samples

Fresh milk samples were collected aseptically from the positive CMT positive quarters (*n* = 488), to detect the prevalence of bacterial isolation in subclinically mastitic quarters, then centrifuged at 3000 rpm for 15 min. The supernatant fluids were discarded, and a loopful from the sediment was taken and cultivated onto 10% sheep blood agar, Mannitol salt agar, Baird-Parker agar, Edward’s medium and MacConkey’s agar (Oxoid, Ltd., Basingstoke, UK) and then incubated at 37 °C for 24–48 hrs. Bacterial species were suspected based on phenotypic characters on culture media. Bacterial smears were made from the colonies and stained with Gram’s stain [21]. Samples had more than three bacterial pathogens were judged as contaminated, counted as negative and excluded from all further studies.

### 2.6. Identification of Bacterial Isolates

Presumptive identification of the bacterial isolates was carried out according to their Gram’s stain morphology and colonial and biochemical characteristics. Identification was confirmed by additional laboratory tests according to Quinn et al., NMC and Waller et al. [21,22,23]. For Gram-negative bacterial isolates, the following tests were used: oxidase, catalase, indole, methyl red, Voges–Proskauer, citrate utilization, urease, H_2_S production on TSI, nitrate reduction and motility tests. Meanwhile, in the case of Gram-positive isolates, the following tests were used: catalase, citrate utilization, urease production and coagulase (for *S. aureus*) while catalase, CAMP, gelatin liquefaction, sodium hippurate and bile aesculin hydrolysis, and sorbitol and arabinose fermentation tests (for streptococci). The appropriate API (Analytical Profile Index) kits (API20E, API-Staph and API-Strep; Oxoid, Basingstoke, UK) was also used to identify *Enterobacteriaceae*, NAS and *Streptococcus* spp. isolates. API strips should only be used to identify pure cultures following the manufacturer’s instructions. The different strains used as a positive control in API kits were completely identified bacterial strains supplied by the Department of Bacteriology, Mycology, and Immunology, Faculty of Veterinary Medicine, Beni-Suef University, Egypt.

### 2.7. Antimicrobial Susceptibility Testing

Two hundred representative bacterial isolates were tested due to limited resources. Fifty isolates from each of *S. aureus*, NAS, *Streptococcus* spp. and *E. coli* were selected and examined for their antimicrobial sensitivity (AMS) to 13 different antimicrobial compounds by disc diffusion test. The tested isolates (*n* = 50) were selected randomly but representatively from single and different mixed infections. The following antimicrobial discs ( Oxoid, Ltd., Basingstoke, UK) were used: ampicillin (10 µg), amoxicillin-clavulanic acid (30 µg), cefoxitin (30 µg), cefotaxime sodium (30 µg), vancomycin (30 µg), levofloxacin (5 µg), ciprofloxacin (5 µg), doxycycline HCl (30 µg), clindamycin (2 µg), gentamicin (10 µg), florfenicol (30 µg) and sulfamethoxazole-trimethoprim (25 µg) and colistin sulfate (10 µg). The phenotypic antimicrobial susceptibility tests were conducted using the disc diffusion method on Muller Hinton agar (Oxoid, Ltd., Basingstoke, UK), and the result was interpreted according to CLSI [24]. The AMS was based on the induced inhibition zones according to breakpoints available in the CLSI [24]. Resistance to three/or more antimicrobials from different antibiotic categories was considered as MDR, according to Chandran et al. [25].

### 2.8. Polymerase Chain Reaction

PCR was applied on sixty MDR bacterial isolates (15 isolates from each *S. aureus*, NAS, *Streptococcus* spp. and *E. coli*). The tested isolates were selected according to the results of antimicrobial susceptibility testing as the highest 15 MDR isolates from each bacterial species were selected to estimate four genes in each bacterial species, including two resistance and two virulence-associated genes. DNA Extraction from samples processed using QIAamp DNA mini kit instructions (Cat. No.51304) (Qiagen GmbH, Hilden, Germany). The primers’ sequences ( Metabion international AG, Planegg, Germany), size of amplified products, temperature, and time conditions of the PCR were illustrated in Table 1. The different strains used as PCR positive controls (*S. aureus*, NAS, *Streptococcus* species and *E. coli*) were completely identified bacterial strains; known to have the specified genes, supplied by the Department of Bacteriology, Mycology, and Immunology, Faculty of Veterinary Medicine, Beni-Suef University, Egypt.

### 2.9. Statistical Analysis

The differences in the SCC, average milk composition and milk parameters were estimated by one-way ANOVA followed by Tukey’s multiple comparisons tests using Sigma stat version 3.5 (Systat Software Inc., London, UK). For all treatments, data are presented as the means ± the standard deviation.

## 3. Results

### 3.1. Prevalence of SCM Based on CMT and SCC

Out of 1060 examined QMSs, 488 quarters (46%) were CMT positive, of which 7.5%, 21.8% and 16.7% were assigned as scores 1, 2 and 3, respectively. The total mean SCC of all quarters was 4.91 × 10^5^ ± 5.1 × 10^3^ cells/mL. Meanwhile, it was 1.48 × 10^5^ ± 6.1 × 10^3^ cells/mL in CMT negative quarters, which were considered healthy quarters, while other CMT positive quarters contained gradually increased SCCs with increasing the score (Table 2). SCC of the 1060 tested quarters showed that 475 quarters (44.8%) were SCM positive as individual QMSs having SCC > 2.50 × 10^5^ cells/mL, which were significantly higher than 585 quarters (55.2%) negative CMT group with SCC < 2.50 × 10^5^ cells/mL (Table 2). Within the positive CMT quarters with scores S2 and S3 had significantly higher SCC compared to the S1score and negative CMT quarters (*p* < 0.05).

### 3.2. Distribution of CMT Score and Milk Composition

Lactose, fat, solid not fat (SNF) and total protein contents were significantly decreased in the milk of quarters with increased CMT scores. Notably, the fat content of the quarter’s milk with S3 CMT score was significantly low compared to the S1 and S2 scores and the negative quarters (2.64 ± 0.09, 3.81 ± 0.14, 4.33 ± 0.10 and 5.34 ± 0.08, respectively. Meanwhile, the ash, salt, milk density and milk freezing points significantly increased along with increased CMT scores (Table 3).

### 3.3. Prevalence of Bacterial Agents Isolated from Subclinically Mastitic QMSs

Out of 488 cultured subclinically mastitic QMSs, 441 samples showed positive bacterial isolation with a prevalence of 90.4%; of which 127 samples (26%) had single isolates while 314 samples (64.3%) showed mixed isolates, meanwhile 47 samples (9.6%) showed negative bacterial isolation; of them, 5 samples were judged as contaminated and excluded from all further studies. A total of 887 bacterial isolates were recovered. Of them, 127 isolates were from single infections, while 760 were from 314 co-infections. The most prevalent bacterial isolates were *E. coli* (49.8%) followed by *S. aureus* (44.9%), *Streptococcus* spp. (44.1%), NAS (37.1%) and finally *Enterococcus* spp. (5.9%) (Table 4). Among NAS isolates (*n* = 181), *S. xylosus* was the most prevalent (*n* = 64; 35.4%) followed by *S. chromogenes* (*n* = 23; 12.7%), *S. epidermidis* (*n* = 22; 12.2%), *S. saprophyticus* (*n* = 20; 11%), *S. haemolyticus* (*n* = 18; 9.9%), *S. cohnii* (*n*= 14; 7.7%), *S. simulans* (*n* = 11; 6.1%), *S. hominis* (*n* = 6; 3.3%) and finally *S. lentus* (*n* = 3; 1.7%). Meanwhile, among *Streptococcus* spp. isolates (*n* = 215), *S. agalactiae* was the most prevalent (*n* = 109; 50.7%) followed by *S. dysgalactiae* (*n* = 83; 38.6%) and finally *S. uberis* (*n* = 23; 10.7%). *Enterococcus* spp. (*n* = 29) were identified as *E. faecium* (*n* = 17; 58.6%) and *E. faecalis* (*n* = 12; 41.4%).

### 3.4. Distribution of Bacterial Pathogens in Correspondence to the SCC and CMT

In single infections (*n* = 127), *S. aureus* infection was the most prevalent, followed by NAS, *E. coli* and *streptococci*. In co-infections (*n* = 314), the co-existence of *S. aureus, E. coli* and *streptococci* occurred most frequently (Table 5).

### 3.5. Antimicrobial Susceptibility Testing of Bacterial Isolates

Results of in-vitro susceptibility tests were represented in Table 6. A high percentage of *S. aureus* isolates were resistant to ampicillin (96%) and cefoxitin (78%), while 56% of them were resistant to amoxicillin-clavulanic acid). On the other hand, most of the isolates were sensitive to ciprofloxacin (74%), vancomycin (72%), levofloxacin (68%), florophenicol (66%) and sulfamethoxazole-trimethoprim and gentamicin (60% for each). Moreover, NAS isolates were mostly resistant to ampicillin (90%) and cefoxitin (76%). Meanwhile, they were highly sensitive to ciprofloxacin (82%), florophenicol (80%), levofloxacin (78%), vancomycin and sulfamethoxazole-trimethoprim (76% for each) and gentamicin (70%). Moreover, a high percentage of *Streptococci* isolates were resistant to gentamicin (96%), ciprofloxacin (92%), ampicillin (90%) and amoxicillin-clavulanic acid (88%). Meanwhile, 56% of them were sensitive to vancomycin and florophenicol (56% for each). Moreover, a high percentage of *E. coli* isolates showed resistance to doxycycline (90%), amoxicillin-clavulanic acid and sulfamethoxazole-trimethoprim (88% for each) ciprofloxacin and florophenicol (84% for each) and gentamicin (72%).

### 3.6. Detection of Resistance and Virulence-Associated Genes in MDR Isolates by PCR

According to the results represented in Table 7, nine *S. aureus* isolates (60%), seven (46.7%) and three isolates (20%) harbored *mec*A, *ica*D and *bla*Z genes, respectively, while these genes were also detected in four (26.7%), eight (53.3%) and one isolate (6.7%) of NAS, respectively. The *hlg* gene was not detected in any staphylococcal isolates. Regarding *Streptococcus* isolates, both *aph*(3’)-IIIa and *cfb* genes were detected in 11 isolates (73.3%), while *hyl* and *vanC−2/3as* genes were found in five (33.3%) and four isolates (26.7%), respectively. All tested *E. coli* isolates harbored *tet*A and *sul*1 genes (100%), while 12 (80%) and nine isolates (60%) harbored *fim*H and *tsh* genes, respectively.

## 4. Discussion

Nowadays, SCM is considered one of the most important diseases affecting the dairy industry, causing significant economic losses in Egypt and worldwide [3,38,39,40,41]. Dairy farmers widely use antibiotic therapy as an essential tool to prevent intramammary infection (IMI), especially before calving, or to cure persistent and chronic udder infections and for dry cow therapy at the end of lactation season in herds with a high prevalence of contagious mastitis [42]. Overuse of antimicrobial compounds in dairy farms resulting in emerging resistance among various bacterial pathogens, which is considered a major public health significance due to the risk of resistance transmission to humans and its influence on the current antimicrobials’ efficacy therapeutic protocols [3]. This study was designed to estimate the prevalence of SCM in three dairy herds in North Upper Egypt and identify the most critical causative pathogenic bacteria. Moreover, the determination of antibiotics susceptibility profiles of the isolated pathogens has been performed to select the MDR isolates that have been directed for PCR to explore the antimicrobial and related virulence genes.

In this study, the estimated prevalence of SCM was 46% and 44.8%, according to CMT and SCC results, respectively. These findings were supported by many earlier studies in Egypt and worldwide [16,38,39,41,43,44,45]. High SCM prevalence in dairy herds might be attributed to poor housing and bedding materials, poor hygienic condition, previous history of mastitis, bad milking practice and contaminated milking machines [2,19,38,46]. Therefore, the whole farming and housing systems and udder health management practices inside dairy farms should be improved to minimize the burden of SCM.

Somatic cell count is a very useful tool for monitoring SCM at individual quarters [47]. However, the current study revealed that mean SCC is quietly high, ranging from 1.48–8.66 × 10^5^ cells/mL of milk, and the number of SCC increased with higher CMT scores. Dos Reis and coworkers agreed with the statement that SCC increases with CMT scores and is responsible for the alteration of milk quantity and composition [22,48]. An SCC of more than 5.00 × 10^5^ cells/mL will result in a loss of 1300 Lbs of milk per cow per year in a dairy farm and increased proteolytic activity and lower fat and protein concentrations in milk [5,22,49]. In the current study, Lactose, fat, SNF and total protein contents significantly decreased in the QMSs with increased CMT score and SCC (*p* ≤ 0.05). Meanwhile, the ash, salt, milk density as well asfreezing point significantly increased along with increased CMT score and SCC (*p* ≤ 0.05). Our findings also found the same results that concentrations of milk composition (lactose, fat, protein, SNF) decreased with higher SCC [47,49]. A somatic cell is affected by stress, stage of lactation, milk management and a load of microorganisms and intramammary infection [47]. Therefore, we should improve the management to lower the SCC in milk and produce safe milk for human consumption.

Regarding the results of bacteriological examination illustrated in Table 4 and Table 5, out of 488 cultured subclinically mastitic QMSs (positive CMT), 441 samples (90.4%) showed positive bacterial isolation, and a total of 887 bacterial isolates were recovered. Single infections were recorded in 127 samples (26%) in which *S. aureus* infection was the most prevalent, followed by NAS, *E. coli* and *streptococci*. Meanwhile, a total of 314 samples (64.3%) showed co-infections, with a total of 760 bacterial isolates, with the co-existence of *S. aureus, E. coli* and *streptococci*. Co-infection could be explained as that either one organism was the etiological factor, and the rest were commensals, or one organism provoked primary infection, and the rest were secondary invaders. The high prevalence of co-infections in our study might be attributed to the poor standard hygienic and managemental practices inside the three dairy farms which permitted the spread of both contagious and environmental infections [2,38]. The effect of poor sanitation and improper hygienic measures inside the three dairy farms under study was clear in the current results of microbial isolation from the examined milk samples as high incidence rate of udder co-infections and high incidence rate of environmental microbes. Most of the farm hygienic practices and parameters like hygienic condition of the milking environment, sanitation of the milk containers, udder and teats cleaning, use of separate towel for each cow and the personal hygiene of the milkers were not fully performed by most of the farm owners. On reviewing the available literature, it seemed that co-infections were higher than single infection in causing mastitis [50]. On the contrary, our results differed from those of Zeinhom et al. [51] who recorded the prevalence of bacterial isolation as 67.7% in subclinically mastitic milk samples recording a higher prevalence of single bacterial infections, 51.6%, than mixed ones, 16.1%.

On the other hand, there were 47 samples (9.6%) that had negative bacterial isolation, of which 5 samples were contaminated, excluded and counted as negative. Negative bacterial isolation might be attributed to many reasons: (1) some microorganisms such as mycoplasma, listeria and fungi need specific culture media which were not used in this study; (2) presence of antibiotic residues may explain falsely negative bacteriological results because the withdrawal time is not respected in our herds; (3) single milk sample may not be sufficiently sensitive, and more than one bacteriological sampling is required to determine whether the quarter is infected or not.

The most frequent bacterial pathogens recovered from SCM of apparently healthy dairy cows were *E. coli*, followed by *S. aureus*, *Streptococcus* spp. and NAS. These findings agreed with previous studies characterizing the major bacteria isolated from SCM cases [18,38,44,51]. The prevalence of *S. aureus* (44.9%) agreed with other studies performed in Egypt (46–48%) [3,16], Ethiopia (43.2–44.9%) [52,53] and Pakistan (49%) [54]. However, the lower prevalences of *S. aureus* were also reported in Egypt (24.4%) [55], Tanzania (5.9%) [56], and Turkey (26.1%) [57]. The high *S. aureus* prevalence might be due to transmission through the use of contaminated milking machines and utensils and contaminated milkers’ hands [2,16,53]. *S. aureus* also can evade and influence the cow’s immune system through the production of various enzymes and toxins that cause damage to the mammary tissue and allow more tissue invasion [3,6]. Furthermore, *S. aureus* can survive on the skin and keratin layer of the teat canal of healthy cows, and can confront phagocytosis [6,58]. This highlights the importance of hygiene and managemental practices inside dairy farms. Moreover, it would be a serious hazard for public health because that mastitic milk is usually added into a bulk milk tank, especially in populations where some people could consume raw milk or non-heat-treated dairy products like yogurt or cheese [55]. The prevalence of NAS was 37.1% which was similar to many studies conducted in Egypt (39.8%) [3] and worldwide, e.g., Poland (31.6%) [59], Ethiopia (31%) [60], and Rwanda (40.2%) [61]. A higher prevalence was recorded in Uganda (54.7%) [62], while lower prevalences were reported in Ethiopia (4.1%) [63] and Italy (9.2%) [64]. Furthermore, among NAS species, *S. xylosus*, *S. chromogenes, S. epidermidis, S. saprophyticus, S. simulans* and *S. hominis* were the most frequent in the SCM, which was in line with many earlier studies [23,65,66]. NAS species distribution differed somewhat between CM, and SCM. *S. saprophyticus* and *S. epidermidis* were more common in SCM. At the same time, *S. hyicus* was more common among CM. Meanwhile, *S. chromogenes* and *S. simulans* were present similarly in both types of mastitis [23]. *S. xylosus* was not known to cause SCM. Still, it was detected in 35.4% of this study, and previous studies said that *S. xylosus* is an underestimated pathogenic NAS in bovine SCM [67]. The control strategy of NAS-related mastitis is quite complicated due to the heterogeneity of this bacterial group. Already 15 NAS species or more were associated with udder infection in cows [3].

*Streptococcus* spp. were represented as 44.1% of isolates, and such result was similar to the previous report from Egypt 51] and worldwide [33,52,68]. Lower prevalences were reported in Uganda (16.2%) [62] and Sri Lanka (3.5%) [69]. Furthermore, *S. agalactiae*, *S. dysgalactiae* and *S. uberis* were the most frequent isolates for bovine SCM among Streptococcus species, and many previous reports supported our study [58,59,63]. *S. agalactiae* is a highly contagious pathogen causing bovine SCM that can survive for a long period within the udder of cows and can be transmitted to healthy cows via poor milking hygiene, contaminated milking machine, utensils and contaminated milkers’ hands [57]. Therefore, the hygiene of the dairy farms should be improved to prevent and control SCM. On the other hand, *S. uberis* and *S. dysgalactiae* were considered environmental pathogen, and their main source is the bedding material [57]. Therefore, clean pastures and dry environments, dry milking machines and utensils, and optimum hygiene should be maintained inside dairy farms to decrease such pathogens’ persistence [70]. Moreover, *E. faecalis* and *E. faecium* were identified. Like *S. uberis* and *S. dysgalactiae*, *Enterococci* are also environmental bacterial pathogens causing bovine mastitis [71]. Smulski et al. isolated *Enterococcus* spp. from cows with mastitis [72], while Gomes et al. considered *E. faecalis* a mastitis-causing pathogen [73].

*Escherichia coli*, the most frequent bacterial isolates in this study, is also considered an environmental pathogen causing SCM. In the current study, the prevalence rate of *E. coli* was higher than previously carried out studies (0.8–16.4%) [38,53,64]. The high prevalence of environmental pathogens suggests management mistakes, including overcrowding, insufficient ventilation, inadequate manure removal and general lack of farm cleanliness and sanitation [18,58,60,68]. All these management practices should be appropriately addressed for lowering environmental pathogens inside dairy farms.

Antimicrobial treatment is an effective therapy for the control of mastitis. In Egypt, a number of antimicrobial agents including β-lactams, aminoglycosides, glycopeptides, tetracyclines, phenicols, fluoroquinolones, lincosamides, polymyxins and sulfonamides have been used to control mastitis. However, the extensive use of antimicrobials has been considered the main cause of AMR accumulation. The present study revealed high resistance of *S. aureus* to ampicillin, cefoxitin and amoxicillin-clavulanic acid. Near similar results were previously recorded [3,16,55,57,74]. Phenotypic susceptibility to cefoxitin was employed for valuing methicillin resistance [3]. High incidences of methicillin resistance are very characteristic in notorious *S. aureus* resulting in limited therapeutic options [75]. *S. aureus* isolates showed resistance against the cephalosporins group (cefotaxime and cefoxitin), but Algammal et al. recorded a moderate sensitivity of *S. aureus* strains to cefotaxime [16]. Usually, cephalosporins group antibiotics are moderately active against *S. aureus* and show stable activity in the presence of β-lactamase enzyme [16]. Moreover, NAS species showed high resistance to penicillins (ampicillin and amoxicillin-clavulanic acid) and cephalosporins (cefoxitin and cefotaxime). Those findings were similar to many previous studies [23,58,65,66]. Production of a β-lactamase enzyme is the most common resistance mechanism in staphylococci [3]. Streptococci revealed high resistance to penicillins, cephalosporins, gentamicin and ciprofloxacin (92%). These findings have similarities with many previous reports [57,76]. *E. coli*- showed high resistance to doxycycline, sulfamethoxazole-trimethoprim, ciprofloxacin, penicillin and florophenicol. These results were agreed with those reported by Verma et al. and Youssif et al. [76,77]. High resistance of *E. coli* is due to misuse of antibiotics as tetracyclines, aminoglycosides and fluoroquinolones used for animal treatment [76].

Antimicrobial resistance is conferred by the presence of resistance genes which can be linked to genetic elements, and the use of a particular antimicrobial can select for resistance not only to its own but also potentially to other different antimicrobials. In the current study, the phenotypic properties of AMR were approved using their genotypic characterization using PCR. In this study, about 46.7% of MDR *S. aureus* had the *bla*Z gene and 60% harbored the *mec*A gene. The methicillin-resistant gene, *mec*A, is an inducible 76-kDa penicillin-binding protein carried on a mobile genetic component termed Staphylococcal Cassette Chromosomes (SCCs) [3]. The acquisition of *mec*A promotes staphylococcal resistance to methicillin and other β-lactams antibiotics [75]. The existence of *mec*A positive MDR *S. aureus* in dairy cows has been reported worldwide in many previous scientific reports and studies [55,78,79]. Interestingly, some studies showed that all *mec*A posing *S. aureus* were also positive for the *bla*Z gene [3]. The coexistence of *mec*A and *bla*Z genes in MDR *S. aureus* of milk and farm environment carries a threat for the consumers, farmworkers and veterinarians. Almost all MDR *S. aureus* are of human origin and transmitted to dairy cows due to poor hygiene and management [55,78]. Moreover, *bla*Z and *mec*A genes were found in 53.3 and 26.7% of MDR NAS, and this finding was in line with few previous studies [65,66]. Moreover, 20 and 6.7% of tested MDR *S. aureus* and NAS, respectively, harbored the virulence gene *ica*D responsible for biofilm production, supporting those obtained by previous studies [80,81]. The *ica*D gene was indicated as one of the most reliable genes for biofilm formation [82]. Biofilm-forming microorganisms become more resistant to opsonophagocytosis and conventional antibiotics [75]. This bacterial tolerance is responsible for the chronic status of the disease [83]. Moreover, biofilm formation can be harmful to host tissues because it can promote lysosomal enzymes’ phagocyte release [84]. Morente et al. [85] highlighted the role of biofilms in the development and transfer of resistance in microbial population by the interactions happening via the biofilm.

The production of cytolytic toxins is the main mechanism deployed by *S. aureus* to target host phagocytes [86]. *S. aureus* secretes various exotoxins invading host cell such as haemolysins which are classified into four different toxins; alpha, beta, gamma and delta encoded by *hla*, *hlb*, *hlg* and *hld* genes, respectively. These toxins have a cytolytic effect inducing lysis of a broad spectrum of cells including erythrocytes, platelets, neutrophils and monocytes [87]. Staphylococcal γ-haemolysin (*hlg*) consists of polypeptides designated as S (slow, *hlg*A or *hlg*C) and F (fast, *hlg*B), which cooperatively lysis target cells, where the S components are suggested to affect cell type susceptibilities to these toxins [86]. Gamma-haemolysin belongs to a group of genes that code for both *hlg*A and *hlg*C as the S (slow) component, or *hlg*B as the F (fast) component, which is located in the core genome [13]. In the current study, the *hlg* gene was not detected in any staphylococcal isolates. Such results agreed with those reported by [88] as the *hlg* gene was not identified in any of *S. aureus* isolates; meanwhile, other haemolysin genes *hla* and *hlb* were detected in 77.3 and 27.5% of the isolates, respectively. On the other hand, this disagreed with Abdel-Tawab et al. [89], who detected the *hlg* gene among 58.3% of *S. aureus* isolates. It was found that the *hlb* gene is associated with the presence of the *hla* gene and the same words apply to a relationship *hlg* gene with *hld* gene, suggesting the possibility of a molecular relationship between them [86]. Therefore, further investigations of the four haemolysins and their genes are still required.

MDR streptococci isolates harbored gene *aph*(3’)-IIIa and *cfb.* Ding et al. also reported similar results [90]. One of the most critical virulence strategies in many mammary gland pathogens is their ability to attach to the host cell’s surface. The expression of these genes plays an essential role in the virulence of *S. agalactiae*. During systemic infections, the CAMP factor (*cfb*) may be released, impairing the host immune response [91]. Moreover, the *hyl* gene was detected in 33.3% of the tested streptococci. The hyaluronidase (*hyl*) gene is essential for streptococci pathogenesis, especially S. agalactiae promoting its dissemination into the host tissue and facilitating host cell invasion [92]. On the other hand, all tested MDR *E. coli* expressed *tet*A and sul1 genes while 80 and 60% *fim*H and *tsh* genes, respectively. The same findings were presented in many conducted studies [13,93]. The adhesion is the first step in the virulence mechanisms of *E. coli*. Bacterial adhesins play an essential role in the adherence of bacteria to host epithelial cells [94]. Pathogenic *E. coli* strains may produce temperature-sensitive haemagglutinin (*tsh*), which is considered one of the essential adhesion factors encoded by a *tsh* gene located mainly in high molecular weight plasmids known as ColV [95]. Moreover, based on the presence of serine protease sites in the *tsh* protein and its ability to degrade hemoglobin [96], *tsh* was suggested to act as a protease on a specific substrate in the early stages of the infection, thus promoting lesion formation. Moreover, the *fim*H gene is one of the fimbria adhesin encoding genes helping the microbe better adhesion the epithelial lining cells and improve its destructive action [97].

The results in the current study revealed a strong association between the phenotypes and genotypes of AMR in different bacteria such as between tetracycline and *tet*A and sulfonamide and *sul*1 in *E. coli* (100% for each), aminoglycosides and *aph(3’)-IIIa* in streptococci (73.3%) and MRSA and *mec*A in *S. aureus* (73.3%). However, interestingly, it was found that some strains possessed phenotypes AMR but did not have the corresponding AMR genes such between β-lactams and *bla*Z in *S. aureus*, vancomycin and *van*C in streptococci. These findings were supported by the results of previous studies describing the associations between resistance phenotypes and resistance genes, as well as between resistance and virulence genes in different bacteria [98,99,100,101,102,103]. The inconsistency of the genotype–phenotype association of AMR could be explained by resistance phenotypes that can be expressed upon the stimulation of many different genetic factors that have not been investigated in this study, and each factor may present a unique epidemiological character [102,104]. Another explanation suggested the possibility of other mechanism(s) such as overexpression of efflux pumps, mutations or modifications in the target sites [100].

## 5. Conclusions

Subclinical mastitis has been reported in Egyptian bovine dairy herds in several studies, causing significant economic losses in the dairy industry in Egypt. In the same context, the current study revealed the prevalence of SCM in 46% and 44.8% of tested samples based on investigations by CMT and SCC, respectively. In spite of this, SCM does not produce visible effects on the udder or milk quality but significantly impacts milk composition and human health. The presence of SCM at a high level in the current study is alarming, and its diagnosis is required to apply proper treatment. The most frequent bacterial pathogens recovered from subclinically mastitic cows were *E. coli*, followed by *S. aureus*, *Streptococcus* spp. and NAS. All isolates revealed a high degree of resistance to the most commonly used antimicrobials. This study highlighted the increased proportion of MDR bacterial pathogens isolated from lactating cows in some Egyptian dairy herds due to the excessive misuse of the antimicrobials. A significant association between the phenotypes and genotypes of AMR in different bacteria was recorded. Extensive farm hygiene and strict milking management practices are required to avoid environmental pathogens and reduce udder infection and mastitis.

## Figures and Tables

**Table 1:** Primers sequences, target genes, amplicon sizes and cycling conditions.

Primers		Primers Sequences	Amplified Product	Primary Denaturation	Amplification (35 cycles)			Final Extension	References
					2ry Denaturation	Annealing	Extension		
Staphylococci	*mec*A	GTAGAAATGACTGAACGTCCGATAA CCAATTCCACATTGTTTCGGTCTAA	310 bp	94 °C 5 min	94 °C 30 s	50 °C 30 s	72 °C 30 s	72 °C 7 min	[26]
	*bla*Z	ACTTCAACACCTGCTGCTTTC TGACCACTTTTATCAGCAACC	173 bp	94 °C 5 min	94 °C 30 s	54 °C 30 s	72 °C 30 s	72 °C 7 min	[27]
	*hlg*	GCCAATCCGTTATTAGAAAATGC CCATAGACGTAGCAACGGAT	937 bp	94 °C 5 min	94 °C 30 s	55 °C 40 s	72 °C 50 s	72 °C 10 min	[28]
	*ica*D	AAACGTAAGAGAGGTGG GGCAATATGATCAAGATA	381 bp	94 °C 5 min	94 °C 30 s	49 °C 45 s	72 °C 45 s	72 °C 10 min	[29]
Streptococci	*aph(3’)-IIIa*	GGCTAAAATGAGAATATCACCGG CTTTAAAAAATCATACAGCTCGCG	523 bp	94 °C 3 min	94 °C 30 s	55 °C 30 s	72 °C 1 min	72 °C 5 min	[30]
	*vanC−2/3 as*	GATTTGTTCTTGCTGGTTGG CAATCGAAGCACTCCAATCATCTCCCT	427 bp	94 °C 3min	94 °C 30 s	56 °C 30 s	72 °C 1 min	72 °C 5 min	[31]
	*hyl*	ACAGAAGAGCTGCAGGAAATG GACTGACGTCCAAGTTTCCAA	276 bp	94 °C 3 min	94 °C 30 s	56 °C 30 s	72 °C 1 min	72 °C 5 min	[32]
	*cfb*	TTTCACCAGCTGTATTAGA GTTCCCTGAACATTATCTT	154 bp	96 °C 5 min	94 °C 30 s	56 °C 30 s	72 °C 1 min	72 °C 5 min	[33]
*E. coli*	*tet*A	GGTTCACTCGAACGACGTCA CTGTCCGACAAGTTGCATGA	576 bp	94 °C 5 min	94 °C 30 s	50 °C 40 s	72 °C 45 s	72 °C 10 min	[34]
	*sul*1	CGGCGTGGGCTACCTGAACG GCCGATCGCGTGAAGTTCCG	433 bp	94 °C 5 min	94 °C 30 s	60 °C 40 s	72 °C 45 s	72 °C 10 min	[35]
	*fim*H	TGCAGAACGGATAAGCCGTGG GCAGTCACCTGCCCTCCGGTA	508 bp	94 °C 5 min	94 °C 30 s	50 °C 40 s	72 °C 40 s	72 °C 10 min	[36]
	*tsh*	GGTGGTGCACTGGAGTGG AGTCCAGCGTGATAGTGG	620 bp	94 °C 5 min	94 °C 30 s	54 °C 40 s	72 °C 40 s	72 °C 10 min	[37]

*mec*A: Methicillin resistance gene. *bla*Z: β-lactams resistance gene. *hlg*: Gamma haemolysin protein gene. *ica*D: Biofilm gene. *aph*(3’)-IIIa: Aminoglycoside resistance gene. *van*C−2/3as: Vancomycin resistance gene. *hyl*: Hyaluronidase gene. *cfb*: CAMP factor gene. *tet*A: Tetracyclines resistance gene. *sul*1: Sulphonamides resistance gene. *fim*H: Fimbria adhesion gene*. tsh*: Temperature-sensitive haemagglutinin gene. 3. Results.

**Table 2:** Frequency distribution of SCC in relation to CMT scores in the examined QMSs.

SCC (×105 cells/mL)	Negative CMT		Positive CMT ^1^								Total QMSs	
			S1		S2		S3		Total			
	No	%	No	%	No	%	No	%	No	%	No	%
< 2.50	547	51.6	30	2.8	8	0.8	0	0	38	3.6	585	55.2
2.50: < 5.00	25	2.4	44	4.0	155	14.6	13	1.2	212	20	237*	22.4
5.00: < 10.00	0	0	6	0.6	57	5.4	99	9.3	162	15.3	162*	15.3
>10.00	0	0	0	0	11	1.0	65	6.1	76	7.2	76*	7.2
Total	572	54	80	7.5	231	21.8	177	16.7	488	46	1060	100
Average SCC ^2^ (×10^5^ cells/mL)	1.48 × 10^5^ ± 6.1 × 10^3a^		2.78 × 10^5^ ± 5.6 × 10^3b^		4.57 × 10^5^ ± 7.1 × 10^3c^		8.66 × 10^5^ ± 7.2 × 10^3d^				4.91 × 10^5^ ± 5.1 × 10^3^	

^1^ %: Were calculated according to the total No. of quarter milk samples (*n* = 1060), S1: score+ ; S2: score++ and S3: score+++ * SCM quarters based on SCC (having SCC > 2.50 × 105 cells/mL with a total of 475 quarters; 44.8%). ^2^ Somatic cell counts followed by different superscript small letters are significantly different (*p* < 0.05).

**Table 3:** CMT scores and milk compositions and some milk parameters.

CMT Results ^1^	Average Milk Compositions (%) ^2^						Milk Parameters ^2^	
	Lactose	Fat	SNF	Total Protein	Ash	Salt	Milk Density	Freezing Point
Negative	5.55 ± 0.20 ^a^	5.34 ± 0.08 ^a^	8.63 ± 0.24 ^a^	4.01 ± 0.12 ^a^	0.60 ± 0.01 ^a^	0.61 ± 0.02 ^a^	25.25 ± 0.53 ^a^	−0.502 ± 0.02 ^a^
S1	4.92 ± 0.05 ^b^	4.33 ± 0.10 ^b^	8.25 ± 0.16 ^b^	3.82 ± 0.09 ^a^	0.64 ± 0.01 ^a^	0.69 ± 0.01 ^b^	27.18 ± 0.87 ^a^	−0.528 ± 0.04 ^b^
S2	4.61 ± 0.01 ^b^	3.81 ± 0.14 ^c^	7.92 ± 0.17 ^b^	3.65 ± 0.03 ^b^	0.69 ± 0.02 ^b^	0.78 ± 0.01 ^c^	29.60 ± 0.26 ^b^	−0.566 ± 0.01 ^c^
S3	4.22 ± 0.05 ^c^	2.64 ± 0.09 ^d^	7.31 ± 0.07 ^c^	3.50 ± 0.06 ^b^	0.76 ± 0.03 ^c^	0.93 ± 0.02 ^d^	32.85 ± 0.18 ^c^	−0.601 ± 0.03 ^d^

^1^ SNF: solid not fat. S1: score+; S2: score++ and S3: score+++. ^2^ Parameters in the same column followed by different superscript small letters are significantly different (*p* ≤ 0.05).

**Table 4:** Prevalence of different bacterial isolates in subclinically mastitic quarters.

Bacterial Isolates	No. of Subclinically Mastitic Quarters	Positive Isolation					
		Single		Co-Infection		Total	
		No	%	No	%	No	%
*E. coli*	488	25	5.1	218	44.7	243	49.8
*S. aureus*		51	10.5	168	34.4	219	44.9
*Streptococcus* spp.		13	2.7	202	41.4	215	44.1
NAS		38	7.8	143	29.3	181	37.1
*Enterococcus* spp.		0	0	29	5.9	29	5.9

%: Percentages were calculated according to total subclinically mastitic quarters (*n* = 488); NAS: Non-aureus staphylococci.

**Table 5:** Distribution of bacterial pathogens in correspondence to the SCC and CMT.

Bacterial Infection		Total No. (%)	S1		S2		S3	
			No (%)	SCC*	No (%)	SCC*	No (%)	SCC*
Single	*S. aureus*	51 (10.5)	7 (1.4)	4.40	13 (2.7)	6.06	31 (6.4)	10.10
	NAS	38 (7.8)	21 (4.3)	2.83	13 (2.7)	4.12	4 (0.8)	9.28
	*Streptococci*	13 (2.7)	7 (1.4)	2.44	5 (1)	4.80	1 (0.2)	8.60
	*E. coli*	25 (5.1)	15 (3.1)	2.33	7 (1.4)	5.44	3 (0.6)	9.68
	**Total single**	**127 (26)**	**50 (10.2)**		**38 (7.8)**		**39 (8)**	
Co-infection	*S. aureus + E. coli + Streptococci*	60 (12.3)	3 (0.6)	2.83	31 (6.4)	5.35	26 (5.3)	11.84
	NAS + *E. coli + Streptococci*	49 (10)	4 (0.8)	2.78	29 (5.9)	5.20	16 (3.3)	6.85
	NAS *+ E. coli*	40 (8.2)	2 (0.4)	2.82	24 (4.9)	5.15	14 (2.9)	8.19
	*S. aureus + Streptococci*	31 (6.4)	-	-	17 (3.5)	4.52	14 (2.9)	8.31
	*S. aureus + Enterococci*	11 (2.3)	-	-	6 (1.2)	4.56	5 (1)	8.37
	*S. aureus + E. coli*	43 (8.8)	2 (0.4)	5.03	30 (6.1)	6.31	11 (2.3)	11.14
	*E. coli + Streptococci*	15 (3.1)	-	-	3 (0.6)	5.53	12 (2.5)	9.23
	*E. coli + Enterococci*	11 (2.3)	-	-	2 (0.4)	5.51	9 (1.8)	8.92
	NAS *+ Streptococci*	24 (4.9)	-	-	15 (3.1)	3.96	9 (1.8)	8.59
	NAS *+ Enterococci*	7 (1.4)	-	-	3 (0.6)	3.91	4 (0.8)	8.52
	*S. aureus +* NAS *+ Streptococci*	23 (4.7)	-	-	14 (2.9)	3.68	9 (1.8)	6.13
	**Total co-infection**	**314 (64.3)**	**11 (2.3)**		**174 (35.7)**		**129 (26.4)**	
**Total bacterial isolation**		**441 (90.4)**	**61 (12.5)**		**212 (43.4)**		**168 (34.4)**	
Negative bacterial isolation		47 (9.6)	19 (3.9)	2.04	19 (3.9)	4.31	9 (1.8)	6.11
Overall total SCM quarters		488 (100)	80 (16.4)	2.78	231(47.3)	4.57	177 (36.3)	8.66

%: Percentages were calculated according to total subclinical mastitis quarters (*n* = 488). * Average SCC of the corresponding no. (×10^5^ cells/mL).

**Table 6:** Results of antimicrobial susceptibility testing of different bacterial isolates.

Class	Antimicrobial Agent	Disc Content (µg)	*S. aureus*			NAS			Streptococci			*E. coli*		
			R	I	S	R	I	S	R	I	S	R	I	S
Penicillins	Ampicillin	10	96	0	4	90	2	8	90	4	6	68	10	22
	Amoxicillin-clavulanic A	30	78	12	10	76	10	14	88	4	8	88	6	6
Cephalosporins	Cefoxitin	30	56	18	26	48	22	30	-	-	-	48	14	38
	Cefotaxime sodium	30	46	12	42	42	16	42	52	8	40	72	6	22
Glycopeptides	Vancomycin	30	16	12	72	14	10	76	36	8	56	-	-	-
Fluoroquinolones	Levofloxacin	5	20	12	68	14	8	78	52	14	34	62	12	26
	Ciprofloxacin	5	16	10	74	12	6	82	92	4	4	84	10	6
Tetracyclines	Doxycycline HCl	30	26	20	54	22	14	64	38	12	50	90	4	6
Lincosamides	Clindamycin	2	28	20	52	30	22	48	46	8	46	-	-	-
Aminoglycosides	Gentamicin	10	24	16	60	18	12	70	96	2	2	72	6	22
Chloramphenicol	Florophenicol	30	24	10	66	14	6	80	36	8	56	84	6	10
Potentiated sulfonamides	Sulfamethoxazole-trimethoprim	25	26	14	60	14	10	76	42	18	40	88	6	6
Polymyxins	Colistin sulphate	10	-	-	-	-	-	-	-	-	-	64	16	20

R = Resistant. S = Sensitive. I = intermediate. %: were calculated according to the No. of tested isolates (*n* = 50).

**Table 7:** Prevalence of resistance and virulence-associated genes among the tested MDR isolates.

Bacterial Isolates	Target Genes	Positive		Negative	
		No.	%	No.	%
*S. aureus* (*n* = 15)	*mec*A	9	60	6	40
	*bla*Z	7	46.7	8	53.3
	*hlg*	0	0	15	100
	*ica*D	3	20	12	80
NAS (*n* = 15)	*mec*A	4	26.7	11	73.3
	*bla*Z	8	53.3	7	46.7
	*hlg*	0	0	15	100
	*ica*D	1	6.7	14	93.3
*Streptococcus* spp. (*n* = 15)	*aph(3’)-IIIa*	11	73.3	4	26.7
	*vanC−2/3 as*	4	26.7	11	73.3
	*hyl*	5	33.3	10	66.7
	*cfb*	11	73.3	4	26.7
*E. coli* (*n* = 15)	*tet*A	15	100	0	0
	*sul*1	15	100	0	0
	*fim*H	12	80	3	20
	*tsh*	9	60	6	40

%: were calculated according to the No. of tested MDR isolates (*n* = 15).
